# Hints for the Sick-Room

**Published:** 1888-09-08

**Authors:** M. M. Basil

**Affiliations:** Author of "The Commoner Accidents to Life and Limb."


### Hints for the Sick-room.

By M. M. Basil, M.A., M.B., C.M., Author of "The Commoner Accidents to Life and Limb."

ADMINISTRATION OF MEDICINES—(*continued*).

The injection of preparations of ergot is to be made in a special manner. It must be thrown deep into the muscles, not merely under the skin. Hence the needle is plunged straight into the tissues for one to two inches or more, care being taken not to snap it. The injection is conveniently made in the gluteal region or in the lower portion of the abdomen.

The needle must be cleaned immediately after the operation, and its wire or bristle introduced to prevent plugging of the lumen. It is best to leave it a few minutes in some clean water, then lift it once or twice with the point upwards. No blowing towards the eye of the needle must be resorted to in the first instance, as such a course is certain to plug up the narrowest part of it by forcing solid portions of matter in that direction. It must be perfectly dry before the wire is introduced. Needles with solid steel points and lateral apertures are often the most satisfactory ones.

Give no information to the patient as to the nature of the fluid used for hypodermic injection, especially if it is morphia. Watch the patient carefully for some time after the injection. Indeed, in every case where sleeping-draughts or any medicines are used to produce striking effects, you must not forget to watch that patient carefully. Important alterations in the human body cannot be produced suddenly without subjecting it to some risks, and it is well to be on the look-out for the supervention of dangers which have been courted.

Lastly, it would be advisable for you not to undertake the hypodermic injection of any fluid whose proper dose is less than ten minims. The safety of no patient should be made dependent on the capability of a nurse to measure exactly a quantity of liquid below this point.

Injection of liquids into the lower bowel is resorted to frequently, and as the process is nearly always carried out by the nurse, it must be considered here in detail. Enemata may be divided into purgative, medicinal, and dietetic classes. This division is not quite logical, because, first, fluids are sometimes injected into the bowel for objects not included under any of these classes; and secondly, some of these divisions overlap each other. But on the whole the classification is a useful one for practical purposes.

Purgative enemata are given to empty the lower bowel. Ingram's syringe is the best form of apparatus to use; the irrigator may also be utilised for the purpose. The enema-tube must be clean, well oiled, and warmed. Every care must be taken not to inject any air either before the liquid commences to enter the bowel or after the whole of it has been nearly injected. When the enema contains oil or turpentine, not held in suspension by an emulsion, but floating freely on the water, it is best to administer the oil first and follow it up with the rest of the injection. Some writers state that such a mixture may be stirred up while it is being injected, but this is by no means satisfactory.

Purgative enemata may consist of warm water or water plus castor oil, olive oil, glycerine, soap, etc. It is essential to remember that the purgative action depends mainly on the quantity and not the quality of the fluid. The purgative enema differs from the dietetic and medicinal form in the quantity of water which goes to constitute it. It is necessary to use 1, 2, 3, or more pints of water at about 85 to 90 degrees Fahr. The water should not be cold, except when the contrary is distinctly expressed; and it should, as a rule, not be of a higher temperature than 90 degrees Fahr. Please remember the general principle that very cold or hot water must not be injected into any of the cavities of the body, such as the rectum, bladder, vagina, uterus, stomach, pleural cavity, or large abscess cavities. When the washing out of any of these cases is in your charge, you must pay particular attention to the temperature of the water; and remember not to present cold or very hot water to the surgeon when he conducts the operation personally. In certain circumstances injections of cold or very hot water are given intentionally, but these are exceptional conditions, and do not interfere with the general principle just mentioned. Enemata are best given in the position which we shall describe later on as that of Marion Sims. If, however, from any cause this position cannot be conveniently assumed, the process may be conducted equally well with the patient lying on one or other side, or on the back. The tube is introduced with the precautions mentioned under suppositories. If there is any the least difficulty in its insertion, it is best to withdraw it and change the direction. It should be coaxed onwards, never pushed unduly.

Should severe griping come on while the liquid is being injected, the action of the syringe must be stopped and recommenced on the cessation of the spasm. If the fluid returns too quickly, the nates may be held together, or the perineum supported gently through a folded towel. In some cases one or two fingers may be introduced into the bowel along the tube, so as to induce forcible contraction of the muscles. The fingers and tube are firmly gripped and no return of the fluid permitted.

Large enemata may generally be administered in suitable cases without risk of doing any harm. There are some conditions, however, where disastrous results may follow the over-distension of the rectum by liquids. In this country pelvic and perineal operations are practically the two classes of cases where especial care is necessary. The rectum can not be distended with impunity after removal of pelvic tumours, the evacuation of large pelvic abscesses, and other similar operations. The walls of the bowel, if involved in the disease, may have been weakened by the operation, and are therefore liable to give way under provocation. Then, again, it is the practice of some surgeons to make sure that the bowels are kept empty by daily evacuation in all cases of operations about the perineum. If this is effected by enemata, the process must be conducted with extreme gentleness, otherwise the aim of the operation may be entirely defeated. In cases of cancer of the rectum or its neighbourhood, in acute dysentery involving the deeper portions of the bowel, in some forms of typhoid fever, and of prolonged diarrhœa, serious accidents may follow the reckless distension of the bowel. Purgative enemata are, however, rarely required in these cases. In strangulated hernia or other forms of intestinal obstruction large enemata must not be given without especial instructions. In certain types of dysentery, both acute and chronic, continuous irrigation of the bowel is an efficient mode of treatment. A double catheter may be used as the rectal tube, and the irrigation conducted by the gravitating action of water, the return-flow tube preventing over-distension of the bowel. It cannot be too widely known that, according to recent experience, the obstinate ulcers of chronic dysentery heal rapidly under continuous irrigation with a solution of nitrate of silver of 20 grains to the pint of water.

It may be well to remember that glycerine is very efficient for relieving obstinate constipation due to hard and impacted fœcal masses. It succeeds where the usual enemata have been tried with no satisfactory results.

*(To be continued.)*

